# Recurrent Melanoma In Situ at the Skin Graft Site of a Prior Malignant Melanoma of the Left Upper Extremity: A Case Report

**DOI:** 10.7759/cureus.85121

**Published:** 2025-05-31

**Authors:** Brandon C Toliver, Megan Johnson, William Wooden

**Affiliations:** 1 Department of Surgery, University of Iowa Hospitals and Clinics, Iowa City, USA; 2 Department of Dermatology, Indiana University School of Medicine, Indianapolis, USA; 3 Department of Plastic and Reconstructive Surgery, Indiana University School of Medicine, Indianapolis, USA

**Keywords:** melanoma, melanoma in situ, plastic and reconstructive surgery, recurrent melanoma, skin grafting

## Abstract

We present the case of a 43-year-old Caucasian, fair-skinned male with a history of multiple malignant melanomas, who presented in February 2024 for excision of a new melanoma arising within a prior skin graft. The graft had been harvested from his thigh to close an excision of a primary malignant melanoma with a depth of 0.73 mm in 2018. Extensive pathologic interrogation, as well as systemic work-up - including positron emission tomography and computed tomography scans - was performed to determine whether this lesion represented local recurrent disease or a new primary melanoma. This workup was consistent with a primary melanoma in situ, which led to the decision to proceed with skin graft resection and full-thickness skin grafting from his lower left abdomen. The entire previous skin graft was excised. Subsequent surgical pathologic examination of the excised skin graft demonstrated no residual malignant melanoma.

This interesting case demonstrates a unique site of primary melanoma occurrence in a split-thickness skin graft. While this may be the result of incomplete tumor resection at the primary operation, the initial excision had wide margins of 2 cm, and pathological examination demonstrated negative margins. Additionally, the skin graft harvest site was carefully selected, as it came from a relatively sun-protected site. This presentation highlights the importance of continued surveillance of melanoma excision sites, warning patients of possible tumor recurrence or new primary occurrence, and setting expectations that incidental findings may occur.

## Introduction

Melanoma is a malignant tumor caused by the accumulation of mutations in melanocytes, the melanin-producing cells located in the basal layer of the epidermis [1]. Although it accounts for only 4% of all skin cancers, it results in 75% of skin cancer-related deaths [1]. Melanoma may develop in previously normal-appearing skin, known as de novo tumors, or from precursor lesions, such as a commonly acquired nevus or a dysplastic nevus [2].

Diagnosis and assessment of melanomas require excisional biopsies to determine tumor depth. Surgical intervention with wide local excision remains the primary treatment. The Breslow depth, or the vertical thickness of the tumor, is the main prognostic factor used to determine appropriate margins and the need for further interventions. Furthermore, staging of melanoma is determined by the tumor, node, and metastasis (TNM) system. Melanoma in situ is described as T0N0M0, as it is defined by the presence of atypical melanocytes without penetration beyond the epidermis and without nodal or systemic metastasis. Interventions to work up concerning lesions may include sentinel lymph node biopsy, skin grafting, or medical therapies for advanced cases [2]. Among all melanoma patients, about one-third will experience recurrence, and most cases occur within 5 to 10 years of primary treatment, at a loco-regional or distant site [3]. 

We present the case of a 43-year-old Caucasian male with recurrence of melanoma in a skin graft overlying a prior wide local excision site for malignant melanoma. 

## Case presentation

We present the case of a 43-year-old male with a history of multiple malignant melanomas, who underwent a wide local excision with 2 cm margins for a malignant melanoma with a Breslow depth of 0.73 mm on the left ventral forearm, with split-thickness skin grafting using a graft harvested from the right proximal anterior thigh at an outside hospital in 2018. In late 2023, he presented to his local dermatologist with concerns about a new lesion within the skin graft site on the left anterior forearm, for which he underwent an excisional biopsy. Pathology revealed broad and asymmetric intraepidermal melanocytic proliferation, limited to the basal layer, with dermal fibrosis extending to the margin. Furthermore, the lesion was diffusely present overlying a cicatrix and focally extended laterally to it, which is consistent with recurrent melanoma in situ. 

**Figure 1:**
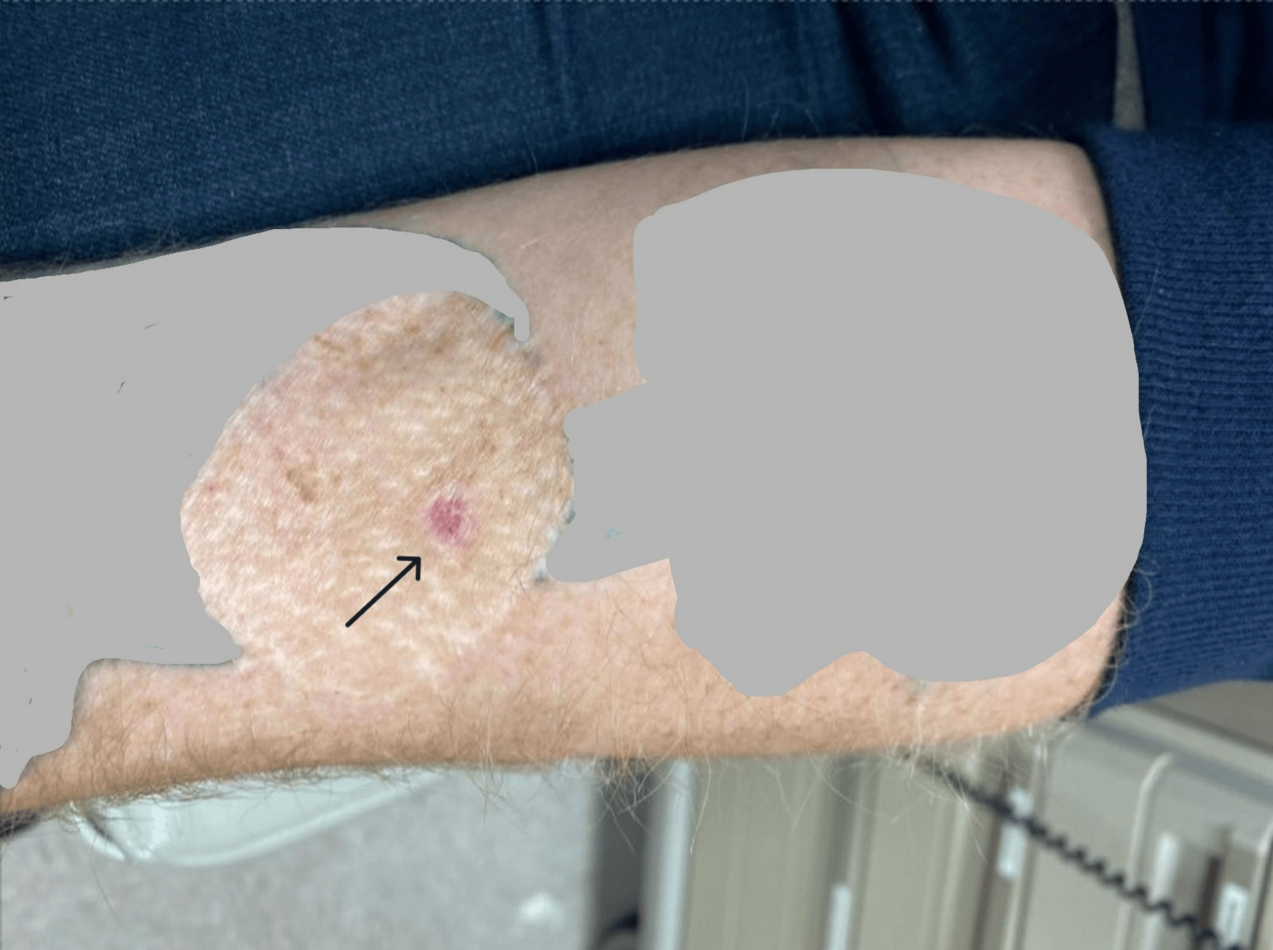
Melanoma in situ status post excision biopsy within skin graft Portions of the image are obscured to omit patient-identifying information. A black arrow demonstrates the lesion of interest. Of note, this shows the appearance of the patient's left ventral forearm after undergoing an excisional biopsy with his dermatologist a few months prior.

He was subsequently referred to our clinic. Our examination did not reveal any clinically significant lymphadenopathy or any additional concerning lesions. Unfortunately, there is no photographic evidence of the lesion prior to the biopsy, but the patient reported it as an atypical mole - without ulceration, bleeding, or causing him pain or itchiness. We visualized the healing biopsy site on the left forearm, as seen in Figure 1. In February 2024, we performed wide local excision, adjacent tissue rearrangement, and full-thickness skin grafting. Resection of the skin graft from the left forearm revealed what appeared to be a venous vascular malformation, separate from the flexor tendons and median nerve. The entirety of the affected skin graft was excised, along with the suspected venous vascular malformation. Preoperatively, we selected a site on the patient’s left lower abdomen, as it was an area protected from the sun, had no visualized lesions or other deformities, and the patient denied having any skin lesions or concerns at that site. Therefore, a full-thickness skin graft was harvested from this site and grafted to the left forearm. 

He followed up with our clinic two weeks after the operation. He was healing well, and the new skin graft appeared to take well, without concerns for seroma, dehiscence, infection, or new lesions. Review of the pathology examination of the excised skin graft and vascular malformation revealed final diagnoses of arteriovenous hemangioma and no residual melanoma. 

## Discussion

This case depicts the presentation of a melanoma in situ within a split-thickness skin graft, performed six years prior, for melanoma in situ, with an incidentally noted arteriovenous hemangioma. 

While this may be the result of incomplete tumor resection at the primary operation, the initial excision - per the outside hospital’s operative note - described margins of 2 cm, and pathological examination demonstrated negative margins. Additionally, the skin graft harvest site was carefully selected, as it came from a relatively sun-protected site - the lower left abdomen - and did not appear to be affected by nevi or other lesions. Though it cannot be stated definitively, it is unlikely that this lesion represents recurrence due to inadequate excision at the prior operation, nor does it seem to be a transplantation of existing disease. 

Although it is not uncommon for patients with melanoma to have de novo lesions or local recurrence, it is rare for melanoma to recur in the skin grafted over a previous excision site. The occurrence of melanoma in transferred tissues is uncommon, and only a handful of cases have been reported. The current literature suggests that these occurrences, or recurrences, have been associated with partial and full-thickness skin grafts used for the management of various diagnoses, including basal cell carcinoma, open scalp defect, compartment syndrome, lymphatic malformation, and melanoma [4-8].

## Conclusions

The development of melanoma in situ in a skin graft is an uncommon phenomenon described in only a few case reports. Various mechanisms have been proposed, but further research is needed to understand this disease process. Our case illustrates the occurrence of a primary melanoma in situ in a split-thickness skin graft harvested from a relatively sun-protected area and grafted to a sun-exposed area of the body - the anterior forearm. Thorough inspection of skin grafts is imperative to prevent transplantation of existing disease, and electing to harvest grafts from sun-protected sites is crucial, although this does not entirely eliminate this risk. This presentation highlights the importance of routine skin surveillance in patients with melanoma; monitoring melanoma excision sites; educating patients about tumor recurrence or primary occurrence; and setting expectations that incidental findings may occur. 
